# Investigation of causal effects of blood metabolites on insomnia and circadian rhythm sleep wake disorders

**DOI:** 10.3389/frsle.2024.1333154

**Published:** 2024-05-03

**Authors:** Zheng Lv, Liyuan Huang, Yongfu Song, Yuejiao Lan, Shizhuo Sun, Yongji Wang, Yinan Ding, Xiaodan Lu

**Affiliations:** ^1^School of Life Science, Changchun Normal University, Changchun, China; ^2^School of Clinical Medicine, Changchun University of Chinese Medicine, Changchun, China; ^3^Precision Medical Center, Jilin Province General Hospital, Changchun, China; ^4^Department of Pediatrics, The Affiliated Hospital to Changchun University of Chinese Medicine, Changchun, China

**Keywords:** sleep disorder, insomnia, circadian rhythm sleep-wake disorders, Mendelian randomization, blood metabolites, causation

## Abstract

**Background:**

Insomnia (IS) and circadian rhythm sleep-wake disorders (CRSWD) are complex disorders with limited and unsatisfactory treatment options that can even cause some side effects. By analyzing blood metabolites to reveal underlying biological processes, studies of sleep and the complex interactions between its influencing factors can be elucidated. Therefore, we hope to bring new hope for the treatment of these diseases through blood metabolites.

**Aims:**

Investigating the causal link between blood metabolites and IS and CRSWD.

**Methods:**

A genome-wide association study (GWAS) for 486 metabolites was used as the exposure, whereas two different GWAS datasets for sleep disorders were the outcome, and all datasets were obtained from publicly available databases. We employed the standard inverse variance weighting (IVW) method for causal analysis, supported by the MR-Egger method, weighted median (WM) method, and MR-PRESSO method for sensitivity analysis to mitigate the impact of pleiotropy. Genetic correlation between IS, CRSWD, and blood metabolites was explored through linkage disequilibrium analysis (LDSC), while Multivariable MR analysis (MVMR) elucidated whether these metabolites exhibit a direct association with IS and CRSWD. Further, we conducted metabolic pathway analysis to identify the specific metabolites driving these relationships.

**Results:**

Employing meticulous MVMR analysis, we have identified specific metabolites that independently influence IS, including 2-hydroxypalmitate (OR 2.95, 95%CI 1.05–8.31 *P* = 0.040), X-11786-Methylcysteine (OR = 0.25, 95%CI 0.08–0.76 *P* = 0.014), and salicylate (OR 0.89, 95%CI 0.83–0.95 *P* = 9 × 10–4). In the context of CRSWD, our findings reveal direct associations with metabolites such as carnitine (OR 0.02, 95%CI: 0.00–0.20, *P* = 0.002), levulinate (OR 0.06, 95%CI: 0.01–0.64, *P* = 0.020), p-cresol sulfate (OR 0.25, 95% CI: 0.09–0.67, *P* = 0.006), and X-14208-Phenylalanylserine (OR 0.36, 95% CI: 0.16–0.81, *P* = 0.014). These discoveries contribute to a nuanced understanding of the distinct metabolic signatures underlying IS and CRSWD.

## 1 Introduction

Insomnia, a common sleep problem where people have trouble falling or staying asleep (Buysse, 2013), has various forms, as classified by the International Classification of Sleep Disorders, Third Edition (Sateia, 2014). Many individuals with insomnia struggle with it for a long time due to different underlying factors (Pavlova, 2017). Some key differences between those with insomnia and those without it include higher metabolic rates during sleep and waking, increased levels of stress hormones like cortisol during early sleep stages, less relaxation in heart rate patterns, and more active brain waves during certain sleep phases. These factors contribute to the complexity of insomnia and its effects on sleep quality (Nofzinger et al., 2004).

The biological clock plays a crucial role in regulating various body functions, including metabolism and our sleep patterns (Gachon et al., 2004). Circadian rhythm sleep-wake disorders (CRSWD) are a group of sleep problems caused by disruptions in our internal body clock or conflicts between our natural rhythms and external cues like light and dark. These disorders often lead to issues like trouble sleeping at night and feeling excessively sleepy during the day, which can really affect a person's quality of life (Sun and Chen, 2022). There are two main types of CRSWD: one where there are long-term changes in how our body clock works, and the other where our sleep schedules clash with our internal rhythms because of changes in our environment (Sateia, 2014). Research has shown that melatonin, a hormone that regulates sleep, can help improve sleep duration and quality, which can make a big difference in how people with CRSWD feel (Nagtegaal et al., 2000).

Currently, common treatments for CRSWD include chronotherapy, phototherapy, and behavioral therapy. It has also been found that melatonin has a certain therapeutic effect on this disease, but it usually relapses after stopping the drug and is accompanied by many side effects (Sun and Chen, 2022). Therefore, the treatment of this disease still requires a more in-depth understanding. If blood metabolites that have an impact on the disease are discovered, we can explore its potential pathogenesis or pathways and provide corresponding treatment options for solving CRSWD.

When people have sleep disorders, their bodies become more active metabolically, leading to changes in the levels of various substances in their blood. These substances, called metabolites, are produced and used up during our body's metabolic processes. Changes in these metabolites can affect both our physical and mental health (Davies et al., 2014). Analyzing blood metabolites could be a valuable tool for spotting diseases early or keeping track of a person's overall health (Tremblay et al., 2019). By understanding these metabolic changes, we might be able to predict and even prevent certain health issues (Zernia et al., 2020). Understanding the connections between different traits and diseases is a big challenge in medical research. Mendelian randomization (MR) analysis is one of the epidemiological research methods that uses genetic variation to link exposure to outcome as an instrumental variable (IV) to assess causality. In contrast to other epidemiologic research strategies, MR can provide unbiased estimates of genotype and is usually immune to confounders and reverse causation. As a result, MR has been widely used in GWAS to aggregate statistics to infer causality for relevant disease risk exposures (Hemani et al., 2018; Zeng et al., 2021; Wang et al., 2022).

Therefore, we hypothesized that blood metabolite profiles could be used to infer causal relationships between blood metabolites and IS with CRSWD. Therefore, we explored the genetic correlations between IS, CRSWD, and blood metabolites by LDSC analysis, and tested whether these metabolites were directly associated with IS and CRSWD by MVMR analysis. In addition, we performed metabolic pathway analysis to investigate the underlying biological processes.

## 2 Materials and methods

### 2.1 Study design

**Figure 1:**
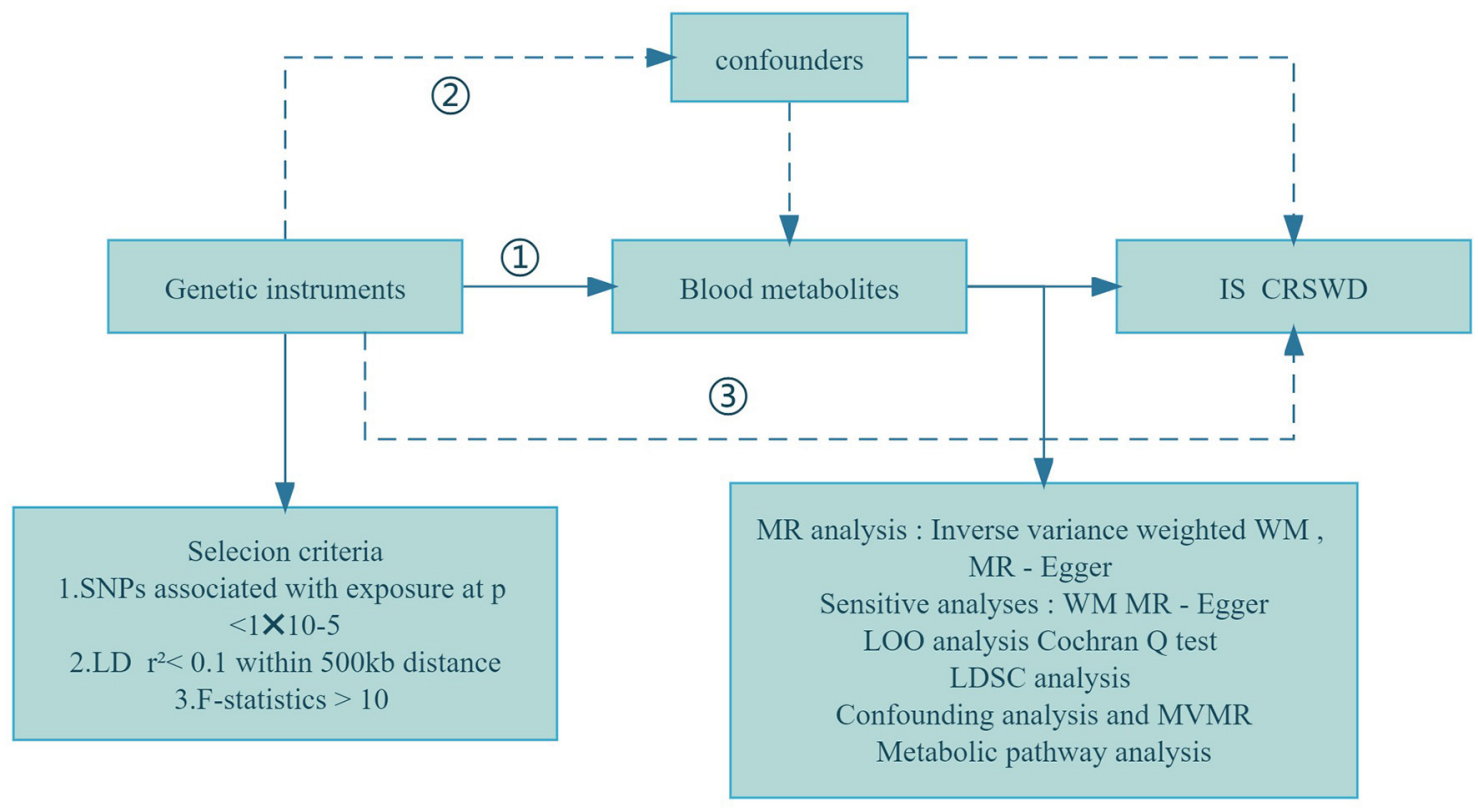
Flowchart of the study design. (1) Genetic tools are strongly correlated with exposure; (2) Genetic tools are independent of confounders; (3) Genetic tools affect outcomes only through exposure. WM, weighted median; LOO analysis, leave-one-out analysis; MR-PRESSO, MR-Pleiotropy RESidual sum and outlier; LD, linkage disequilibrium; LDSC, linkage disequilibrium score; MR-PRESSO, MR-Pleiotropy RESidual sum and outlier; MVMR, multivariable Mendelian randomization analysis; SNPs, single nucleotide polymorphisms.

MR studies rely on selecting specific genetic variations as instrumental variables (IVs), guided by key assumptions: (i) a connection between genotype and exposure exists; (ii) the impact of genotypes on outcomes occurs solely through the mediation of exposure; and (iii) confounding factors are effectively accounted for (Taylor et al., 2014; Boef et al., 2015). All MR analyses were performed using R Studio (version 4.3.1) with the primary R package, TwoSampleMR (Yun et al., 2023). Our study utilized genome-wide association study (GWAS) data to conduct MR analysis, employing blood metabolites as exposure variables and investigating their links to ischemic stroke (IS) and circadian rhythm sleep-wake disorders (CRSWD) as outcomes. The overall process is outlined in Figure 1.

### 2.2 GWAS data for blood metabolites and IS and CRSWD

In our research, we harnessed genetic data related to blood metabolites, accessible from the Metabolomics GWAS server (https://metabolomics.helmholtz-muenchen.de/gwas/). This association was achieved through widespread deployment of genome-wide association scans with high-throughput metabolic profiling, curated by Shin et al., as they combined data on gene expression, heritability, overlap with known drug targets, and previous association with complex diseases. Combined with information on the association with inborn errors of metabolism, the *in vivo* blueprint of metabolism in the human blood has been broadly described (Shin et al., 2014). Details of these metabolites are set out in Supplementary Table 1. We had access to a wealth of valuable genome-wide associations, encompassing 145 metabolic traits and their relationships with 486 distinct metabolites in human blood. This extensive dataset boasted nearly 2.1 million SNPs available for meticulous examination. The 486 serum metabolites were categorized into two overarching groups. The first group comprised known metabolites, rigorously identified through chemical characterization and further classified into eight subtypes: amino acids, carbohydrates, cofactors and vitamins, energy-related compounds, lipids, nucleotides, peptides, and heterometabolism (Kanehisa et al., 2012). The second group encompassed metabolites categorized as “unknown” due to their unspecified chemical properties, distinguished by an “X-” label. Our study incorporated pertinent GWAS data from the dataset published by the Finnish Consortium R9. The cohort for IS consisted of 375,359 individuals, with 4,214 cases and 371,145 controls. For CRSWD, there were 371,145 individuals, with 410 cases mirroring the control group observed in the IS cohort.

### 2.3 MR analysis of IVs selection

The process of selecting instrumental variables demands meticulous screening to ensure the robustness of analyses. In our study, we evaluated the abundance of single nucleotide polymorphisms (SNPs) associated with blood metabolites, ultimately establishing a stringent threshold of 0.05 for inclusion. Simultaneously, we employed the criteria established by Yang and Choi et al. to eliminate linkage disequilibrium LD biases (R^2^ > 0.1 within 500 kb) originating from causal effects (Choi et al., 2019; Yang et al., 2020). Furthermore, we conducted F-value statistics for each SNP, classifying those with F-values < 10 as adverse genetic variations (Burgess et al., 2013). Consequently, this subset of data was meticulously excluded from our analysis.

### 2.4 MR analyses and sensitivity analysis

Drawing from the insights of Burgess et al., we chose the Inverse Variance Weighted (IVW) analysis as our primary method for Mendelian randomization (MR). This method helps us determine the causal link between genetically determined serum metabolite levels and neuroticism. IVW is based on the assumption that there is no horizontal pleiotropy for all SNPs. Under this premise, IVW provides the most accurate assessment of causal effects (Pierce and Burgess, 2013). IVW analysis is considered reliable for assessing causal exposure effects when each instrumental variable meets specific assumptions (P-IVW < 0.05). Additionally, we supplemented our analysis with MR-Egger and weighted median (WM) methods to validate significant estimates of metabolites, ensuring consistency across these three MR approaches (Bowden et al., 2015). To ensure the reliability of our findings, we conducted a thorough investigation into potential pleiotropy and heterogeneity, employing sensitivity analyses. This comprehensive assessment included various tests such as the Cochran-Q test, MR-Egger intercept, Leave-One-Out (LOO) analysis, and MR-PRESSO (Cohen et al., 2015). A significant result in the Cochran-Q test (*p* < 0.05) indicated the presence of heterogeneity, while horizontal pleiotropy was evaluated through the MR-Egger intercept. Furthermore, LOO analysis helped us understand the influence of individual single-nucleotide polymorphisms (SNPs) on outcomes (Burgess, 2017). To enhance the robustness of our statistical evaluation, we utilized an online resource (https://shiny.cnsgenomics.com/mRnd/) to assess the performance of our analyses, maintaining a Type I error rate of 0.05. Power calculations were based on the R^2^ of instrumental variables, the proportion of cases with the outcome, and the odds ratio (OR) obtained from IVW analysis. Metabolites with power values below 0.8 were systematically excluded from our analysis (Brion et al., 2013).

### 2.5 Genetic correlation and direction verification

Expanding on our previous investigations, where we excluded single-nucleotide polymorphisms (SNPs) directly linked to ischemic stroke (IS) and circadian rhythm sleep-wake disorders (CRSWD), we acknowledge the possibility of SNPs unrelated to these disorders influencing their heritability (O'Connor and Price, 2018). To ensure the credibility of our findings and maintain clarity regarding causal effects, we utilized LDSC to examine the genetic correlation between the identified metabolites and the diseases.

### 2.6 Confounding factors and multivariate MR analysis

Confounding factors wield significant influence on study outcomes, often introducing complexities when disentangling the true effects of exposure. To address this challenge, we harnessed an online resource (http://www.phenoscanner.medschl.cam.ac.uk/) to systematically assess the potential confounding effects of 486 metabolite-associated SNPs. Parameters adhered to default settings (Catalog: Diseases & traits, *p*-value: 1E-5, Proxies: None, r^2^: 0.8, Build: 37). This comprehensive step fortified our analytical framework by mitigating the potential interference of confounders on the exposure-outcome relationship. IS is influenced by various risk factors, including age, sex, and a family history of insomnia (Morin and Jarrin, 2022). On the other hand, individuals with neuropathological conditions affecting the hypothalamus, retina, and optic nerve are predisposed to CRSWD (Zee and Abbott, 2020). In the presence of SNPs associated with these confounders, a deliberate process of removal was implemented. Subsequently, MR analyses were conducted to ensure the credibility of the results. This approach was instrumental in isolating the direct effects of each exposure on the outcomes, allowing for the disentanglement of multiple risk factors. MVMR was employed for this purpose, which was further complemented by the IVW method. The utilization of MR-PRESSO enabled the identification and removal of outliers, critical in assessing the presence of pleiotropy in our MR analyses (Burgess and Thompson, 2015; Verbanck et al., 2018; Sanderson, 2021).

### 2.7 Metabolic pathway analysis

To unravel the intricate biological mechanisms through which blood metabolites exert their impact on SD, we conducted metabolic pathway analysis using known metabolites. This comprehensive analysis was based on the KEGG database and was executed with the aid of MetaboAnalyst 5.0 (https://www.metaboanalyst.ca/). By scrutinizing established metabolites and their interconnected pathways, we aimed to shed light on the underlying molecular processes that contribute to Sleep Disorders.

## 3 Result

### 3.1 MR analyses and sensitivity analysis

**Figure 2:**
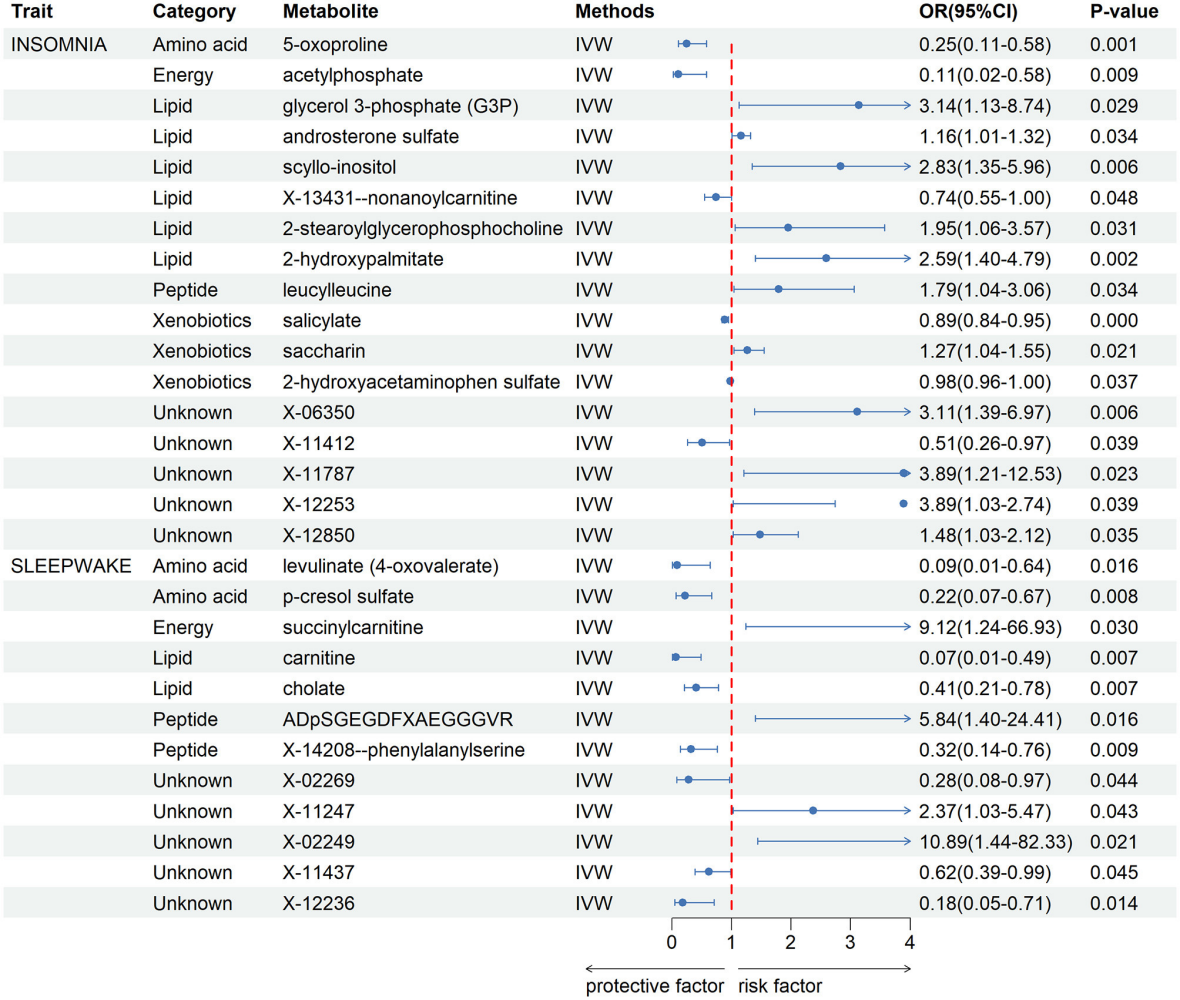
Forest plot for the causality of blood metabolites on IS and CRSWD from inverse variance weighted (IVW) analysis. CI, confidence interval; IVW, inverse variance weighted; OR, odds ratio.

After a rigorous instrumental variable screening procedure, we performed MR Analysis of blood metabolites in 486, see Supplementary Table 2 for detailed data. IVW analysis results preliminarily screened out 17 metabolites with potential causal relationship with IS. After combining complementarity, sensitivity analysis, and calculated power values, 12 metabolites were identified as candidates, including 5-oxoproline (*P* = 0.001), salicylate (*P* = 9 × 10–4), glycerol 3-phosphate (*P* = 0.029), acetylphosphate (*P* = 0.009), saccharin (*P* = 0.021), androsterone sulfate (*P* = 0.034), scyllo-inositol (*P* = 0.006), 2-hydroxyacetaminophen sulfate (*P* = 0.037), X-13431-nonanoylcarnitine (*P* = 0.048), 2-stearoylglycerophosphocholine (*P* = 0.031), 2-hydroxypalmitate (*P* = 0.002), leucylleucine (*P* = 0.034). Seven metabolites that met the criteria were identified in CRSWD, including carnitine (*P* = 0.007), levulinate (*P* = 0.016), cholate (*P* = 0.007), ADpSGEGDFXAEGGGVR (*P* = 0.016), p-cresol sulfate (*P* = 0.008), X-14208—phenylalanylserine (*P* = 0.009), succinylcarnitine (*P* = 0.030).All metabolites have Power values greater than 0.8, and these metabolites will be analyzed in more depth Detailed data of metabolites are shown in Figure 2. The direction and magnitude of the IVW, MR Egger, and WM estimates are consistent. All metabolites have Power values >0.8, and these metabolites will be analyzed in more depth. Detailed results of sensitivity analysis are shown in Supplementary Table 3, and detailed results of MR-PRESSO are shown in Supplementary Table 4. The results of the LOO analysis are presented in Supplementary Figure 1.

### 3.2 Genetic correlation and direction verification

Our LDSC results showed that the metabolite “X-14473” may be genetically correlated with CRSWD (Rg = 1.063, Se = 0.493, *P* = 0.031), but because its chemistry is unknown, more detailed exploration is needed. Detailed results of all metabolites are presented in Supplementary Table 5. At the same time, reverse MR Results (Supplementary Table 6), it also showed that there was no mutual causal relationship between these candidate metabolites and IS and CRSWD.

### 3.3 Confounding factors and multivariate analysis

**Figure 3:**
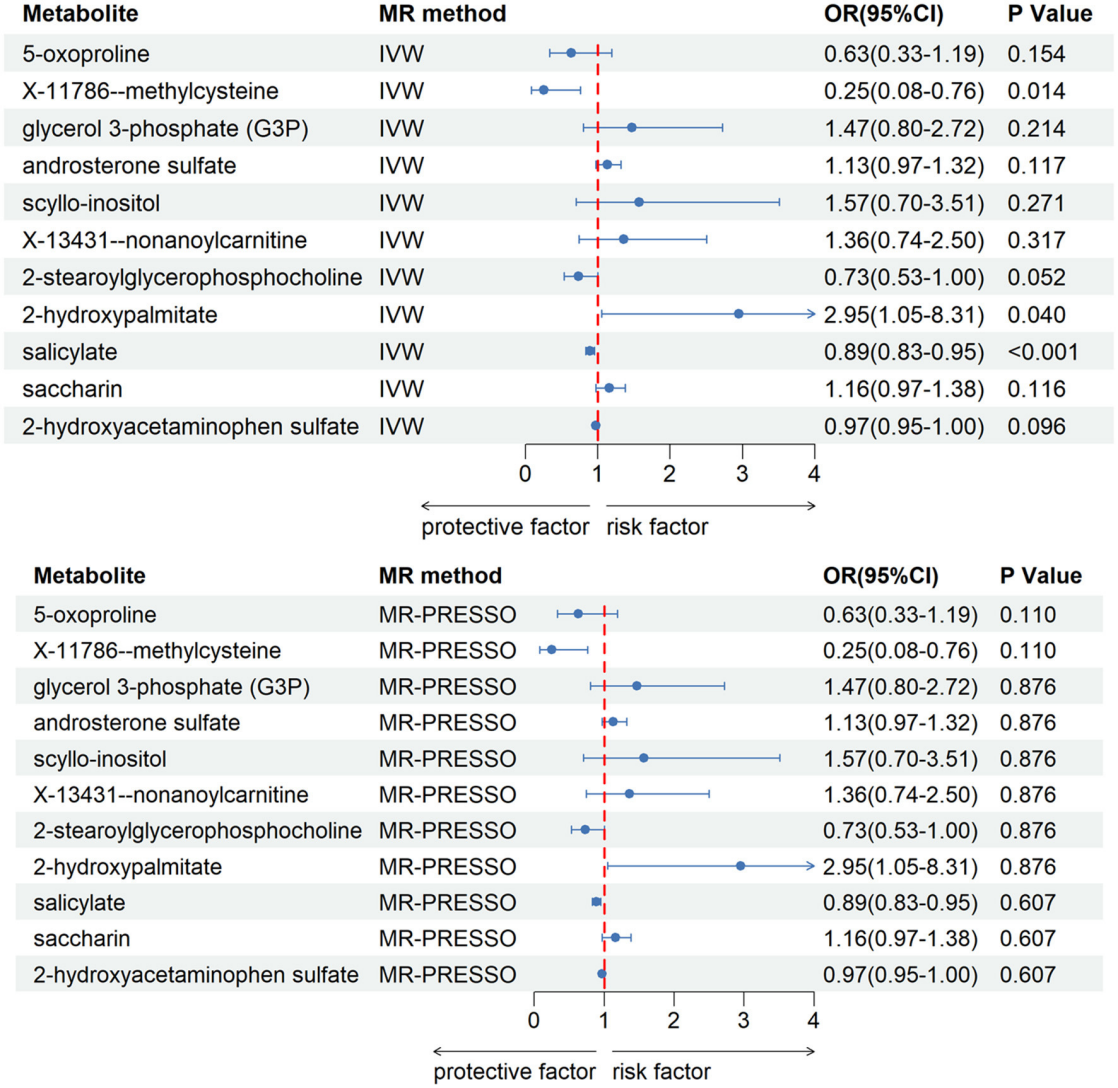
Multivariable MR analysis of the final identified blood metabolites for IS. 95% CI, 95% confidence interval; IVW, inverse variance weighted; MR-PRESSO, MR-Pleiotropy RESidual Sum and Outlier; OR, odds ratio.

**Figure 4:**
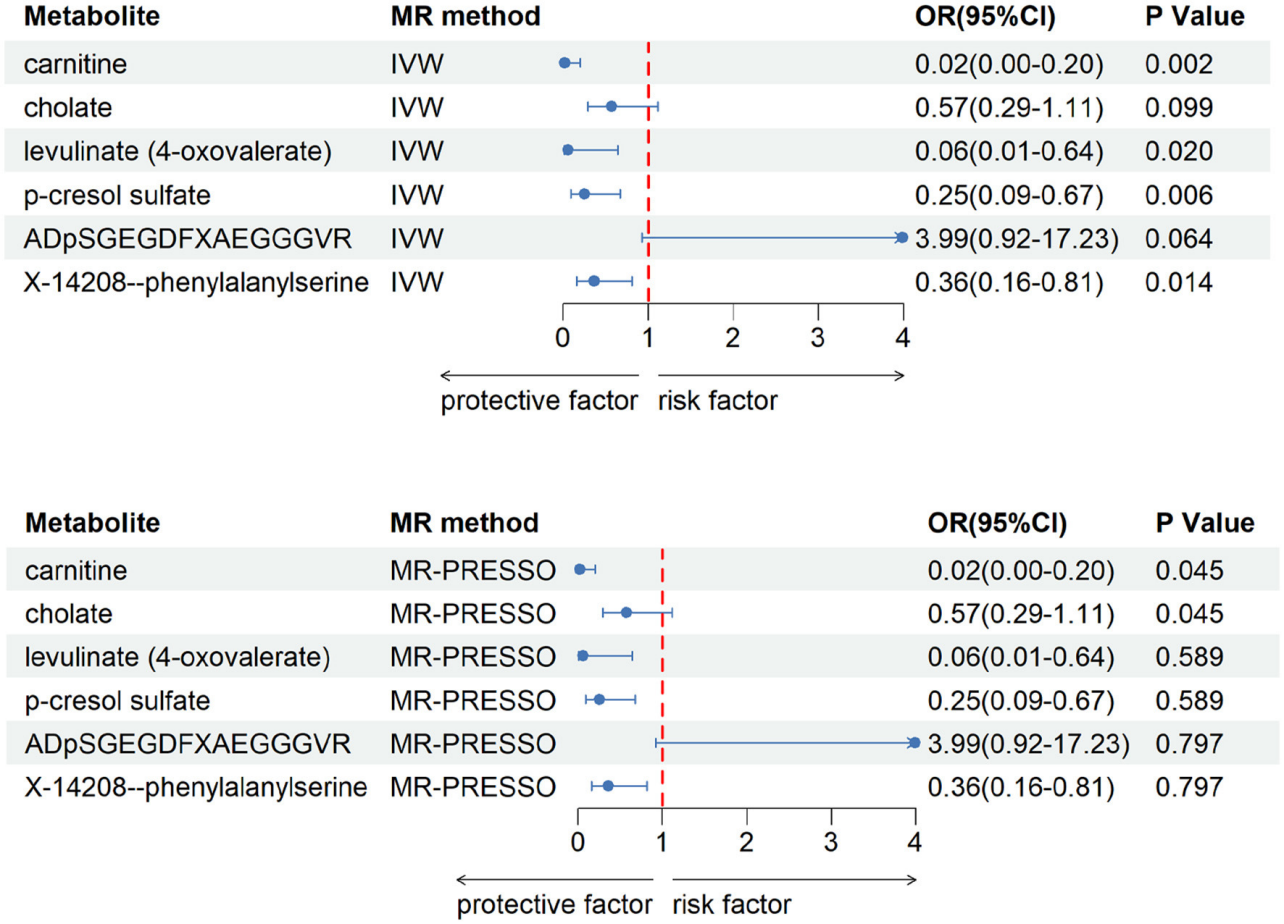
Multivariable MR analysis of the final identified blood metabolites for CRWSD. 95% CI, 95% confidence interval; IVW, inverse variance weighted; MR-PRESSO, MR-Pleiotropy RESidual Sum and Outlier; OR, odds ratio.

After our verification, all metabolites are independent of confounding factors. To verify the direct impact of these candidate metabolites on the disease, we used MVMR analysis, IVW and MR-PRESSO analysis, and found that three metabolites can directly affect IS (Figure 3), including X-11786 methylcysteine (*P* = 0.014), 2-hydroxylaminate (*P* = 0.040), and salicylate (*P* = 9 × 10–4). Four metabolites can directly affect CRSWD (Figure 4), include carnitine (*P* = 0.002), levulinate (*P* = 0.020), p-cresol sulfate (*P* = 0.006), and X-14208—phenylalanylserine (*P* = 0.014).

### 3.4 Metabolic pathway analysis

Through a strict screening process of metabolites (*p* < 0.05) we found that, in IS, 1-acyl-sn-glycerol-3-phosphatecholine and sn-3-phosphoglycerol play important roles in the glycerol phospholipid metabolism pathway, and sn-3-phosphoglycerol is also involved in the glycerol ester metabolism pathway. In CRSWD, L-serine and glycine are involved in the metabolic pathways of glyoxylate and dicarboxylic acid, as well as in the metabolic pathways of glycine, serine, and threonine. Glycine and cholic acid are found to be involved in the biosynthesis pathway of primary bile acids. In the biosynthesis pathway of aminoacyl tRNA, glycine and L-serine act. Sulfate was found to participate in the thiometabolism pathway. In the metabolic pathway of methyl butyrate, butyric acid is the main contributor. Detailed data on metabolic pathways are presented in Supplementary Table 7.

## 4 Discussion

Our MR study, leveraging two distinct GWAS datasets, offers a thorough investigation into the causal relationship between IS, CRSWD, and blood metabolites. The study's primary objective is to illuminate the intricate biological processes and metabolic pathways connecting these disorders with blood metabolites, fostering a more profound understanding of their interplay.

In the IS group, the results of multivariate analysis showed that 2-hydroxypalmitate may directly contribute to the development of insomnia. 2-Hydroxypalmitate and its derivatives have been studied for various potential applications, including in the pharmaceutical and cosmetic industries. They may play a role in biological processes such as cell signaling pathways, lipid metabolism, and inflammation regulation. This metabolite's impact is intimately linked to the HAOX2 gene, primarily expressed in the liver and kidneys, known for its robust activity against 2-hydroxypalmitate (Jones et al., 2000). The specific metabolic pathways and mechanisms of 2-hydroxypalmitate in the kidney may be a key step in solving the mystery of insomnia (Ritter et al., 2016). It is therefore reasonable to speculate that the degree of expression of 2-hydroxypalmitate in the kidneys has some association with insomnia, and that it may be involved as a substrate or regulator of enzymes involved in lipid metabolism and cellular signaling in some of the links between insomnia due to renal disease. In other words, if we can find out in the kidneys of insomnia patients how 2-hydroxypalmitate specifically affects insomnia, it may add some new insights into insomnia. Salicylate was also found to be one of the metabolites that independently affects insomnia in our multivariate analysis results. Salicylates, including salicylic acid found in medications such as aspirin, have been linked to potential effects on sleep. Some people may experience increased alertness or difficulty falling asleep after consuming products containing salicylates. This effect may be due to the stimulant properties of salicylates, especially at higher doses. Salicylates can irritate the gastrointestinal tract in sensitive individuals, causing discomfort or digestive problems and may disrupt sleep. Some people may have specific sensitivities or allergies to salicylates, which may cause symptoms such as headaches, which may affect sleep quality. It has been shown that salicylate-induced tinnitus and consequently insomnia can be alleviated by the protection of overactive nerves by MK-801 (also known as Dizocilpine, a pore blocker of glutamate receptors) (Ritter et al., 2016). This suggests that salicylates can have an effect on insomnia to some extent, and aspirin is one of the common medications that contain salicylic acid, which is often used to prevent diseases such as heart disease, stroke and blood clots. Therefore reducing the intake of such drugs may improve insomnia in some people. The compound X-11786-Methylcysteine does not appear to have been proven in many papers or articles, and its link to insomnia is unclear.

Our research has illuminated the central role of glycerol-3-phosphate (G3P), a blood metabolite, in the “glycerophospholipid metabolism” pathway. G3P emerges as a crucial participant, profoundly impacting the growth and physiological processes of prokaryotes, plants, animals, and humans (Chanda et al., 2011). Other research, led by Zhou and colleagues, highlights G3P's role in processes related to phosphates in the kidneys, particularly concerning renal glycolysis (Zhou et al., 2023). Considering that insomnia is associated with increased arousal and metabolic activity, which leads to more sugar breakdown during the night, our research suggests that G3P might be involved in the changes in glucose levels seen in insomnia cases. This idea is supported by a study conducted by Philip and his team (Gehrman et al., 2018). While these observations offer a compelling perspective, further comprehensive research is necessary to unveil the intricate relationship between G3P and the glucose changes linked to insomnia.

In the CRSWD group, our MVMR analysis identified p-cresol sulfate (PCS) as a metabolite that may have a significant impact on CRSWD.PCS are known as uremic toxins and accumulate significantly in the organs of patients with chronic kidney disease (CKD). These toxins have the potential to induce inflammatory responses, exacerbate oxidative stress, induce glomerulosclerosis and interstitial fibrosis, and exacerbate renal failure. In addition, they play a key role in cardiovascular function (Liu and Tomino, 2018). Hypoxia-induced renal injury is one of the consequences of CRSWD (Xie et al., 2020), which can lead to excessive daytime sleepiness and may trigger respiratory problems such as dyspnea and hypoxia. It can be hypothesized then that the symptoms of CRSWD, which are caused by impaired renal or cardiovascular function and consequently, can be ameliorated to a certain extent by removing or reducing the accumulated PSC in the body. Carnitine is involved in fatty acid metabolism for energy production. Disruption of metabolic pathways, including those involving carnitine, may affect the regulation of circadian rhythms. Carnitine can be obtained from dietary sources, particularly animal products such as meat and fish. Dietary factors can affect overall health and wellbeing, including sleep quality and circadian rhythms. L-carnitine may be present in certain products or environments and may indirectly affect sleep patterns. Therefore, if products containing L-carnitine (e.g., food additives) trigger an allergic reaction or intolerance in some individuals, resulting in discomfort or other symptoms, or if the proportion of meat and fish in the diet is increased, it may interfere with the quality of sleep and lead to the development of CRSWD.

Whereas levulinate does not seem to find a relevant pathway in CRSWD, it is more often used in catalytic oxidation, biorefineries, etc. The same is true for X-14208-Phenylalanylserine, for which we have not found a relevant use at this time due to undefined chemical properties.

Research on PCS-related treatments may represent a pivotal approach to addressing insomnia within this specific population. Additionally, our investigation reveals glycine's involvement in four metabolic pathways, namely “glyoxylate and dicarboxylic acid metabolism,” “glycine, serine, and threonine metabolism,” “aminoacyl tRNA biosynthesis,” and “primary bile acid biosynthesis.” This finding instigates keen interest in the potential relationship between glycine and CRSWD. Glycine, the simplest and most vital amino acid in the human body, is chiefly synthesized in the liver and kidneys. It serves as a precursor for the synthesis of essential compounds, including collagen, creatine, glucose, and purine. Glycine also participates in immune functions, anti-inflammatory processes, and antioxidant reactions (Imenshahidi and Hossenzadeh, 2022). Studies indicate a profound association between circadian rhythms and hormone secretion, temperature fluctuations, and melatonin levels. Melatonin's role in regulating circadian rhythm-related genes, such as PER1, PER2, and BMAL1, is well-documented (Pavlova, 2017). Moreover, the depletion of glycine in mice, stemming from PER2 gene knockout, leads to intestinal metabolic disorders and potential alterations in liver antioxidant and inflammatory responses (Zhen et al., 2022). Recent research highlights the nighttime reduction in the number and function of β2-adrenergic receptors, linked to a genetic polymorphism involving glycine 16 substitution (Martin, 1998). These insights may offer valuable avenues for future exploration, suggesting that glycine could potentially affect renal function through specific metabolic pathways, contributing to the emergence of CRSWD. However, further investigations are imperative to elucidate these intricate mechanisms.

Our study offers crucial insights into the intricate relationship between blood metabolites and two distinct sleep disorder phenotypes, namely IS and CRSWD. However, we acknowledge certain limitations within our research. Firstly, the utilization of GWAS data from the European population restricts the generalizability of our MR results. Consequently, a more comprehensive validation in a broader population would substantially bolster the credibility of our findings. Secondly, this article is an exploratory study, aiming to discover as many potential positive results as possible, so multiple testing correlation is not performed. Finally, while our MR analysis has successfully identified links between several metabolites and IS and CRSWD, further detailed and comprehensive investigations are imperative to ascertain the efficacy of these discoveries in influencing the course of these diseases. In light of these considerations, our research serves as a stepping stone, urging the scientific community to engage in deeper, more extensive explorations of this complex interplay.

## Data availability statement

The datasets presented in this study can be found in online repositories. The names of the repository/repositories and accession number(s) can be found in the article/Supplementary material.

## Author contributions

ZL: Conceptualization, Data curation, Investigation, Methodology, Resources, Software, Supervision, Validation, Visualization, Writing – original draft. LH: Data curation, Investigation, Methodology, Software, Validation, Writing – review & editing. YS: Data curation, Methodology, Software, Validation, Writing – review & editing. YL: Resources, Supervision, Writing – review & editing. SS: Resources, Software, Supervision, Writing – review & editing. YW: Investigation, Project administration, Resources, Supervision, Writing – review & editing. YD: Project administration, Resources, Supervision, Writing – review & editing. XL: Funding acquisition, Project administration, Resources, Supervision, Writing – review & editing.
