# Supplementary material for: Investigation of causal effects of blood metabolites on insomnia and circadian rhythm sleep wake disorders
Source: Front Sleep. 2024 May 3;3:1333154. doi: 10.3389/frsle.2024.1333154 (PMC12713914; doi:10.3389/frsle.2024.1333154)
Supplement: Supplementary file 1 [file Data_Sheet_1.docx]

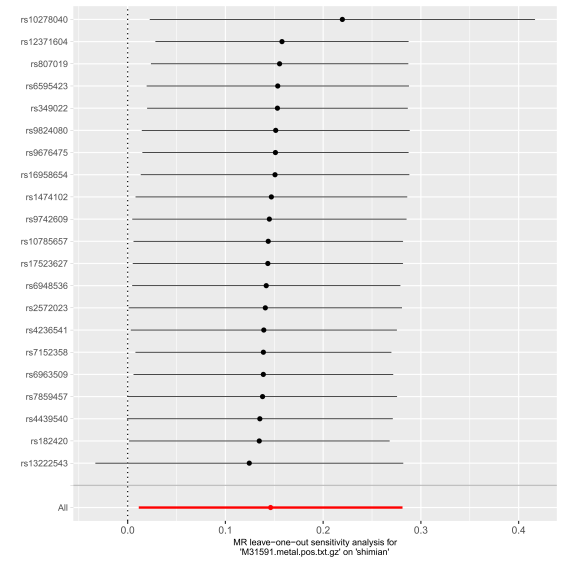

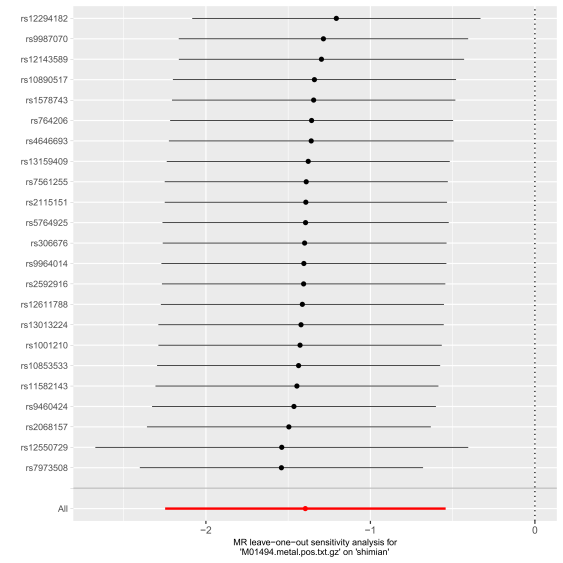

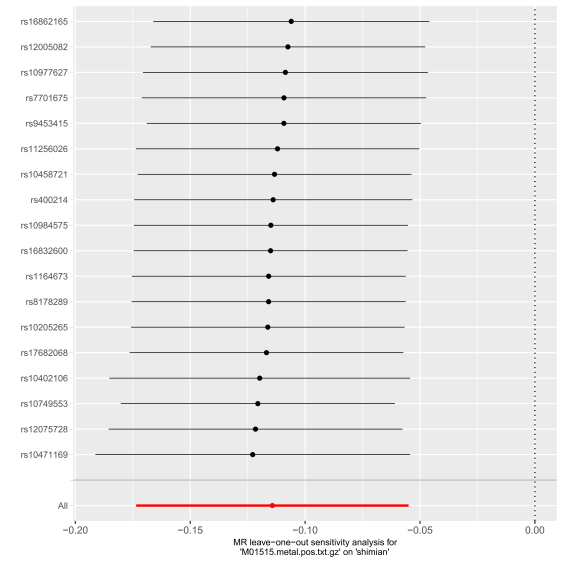

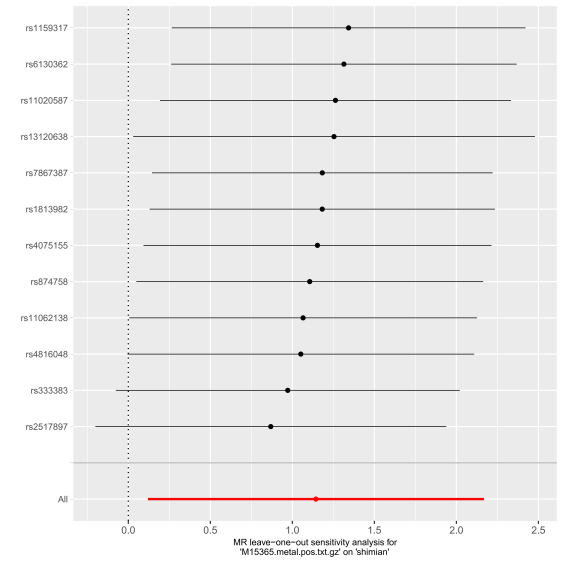

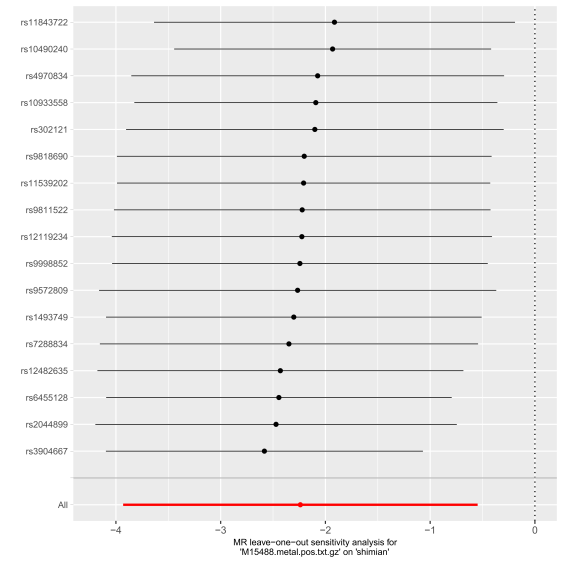

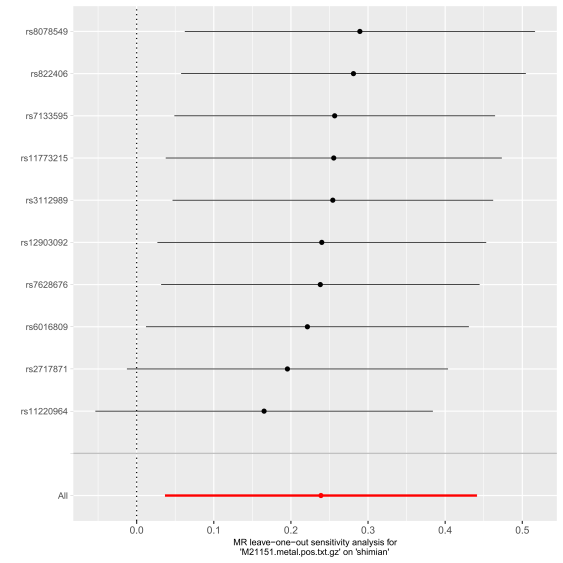


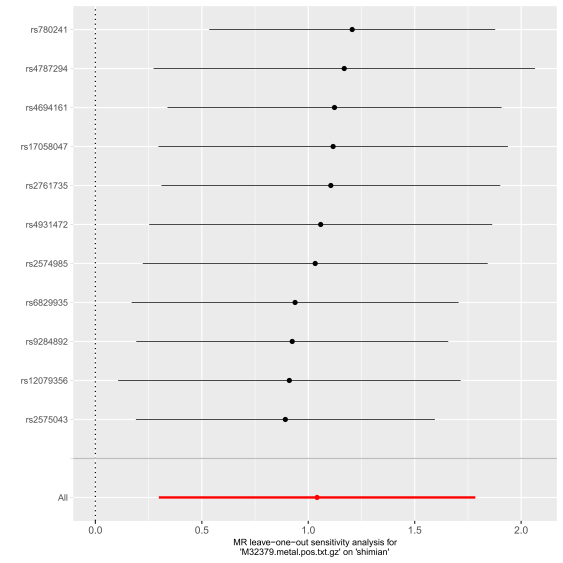

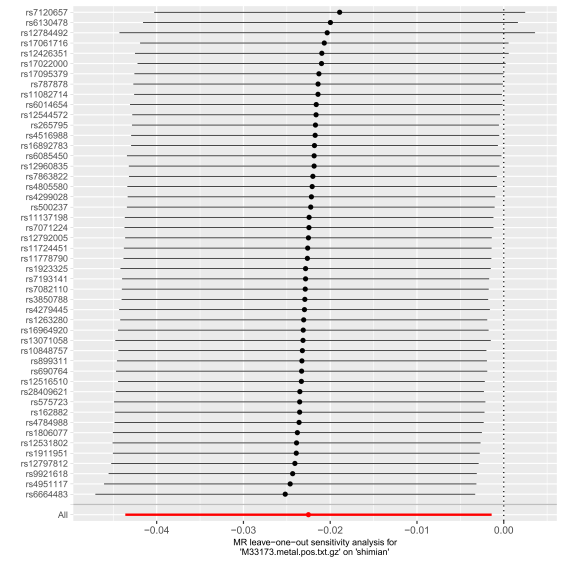

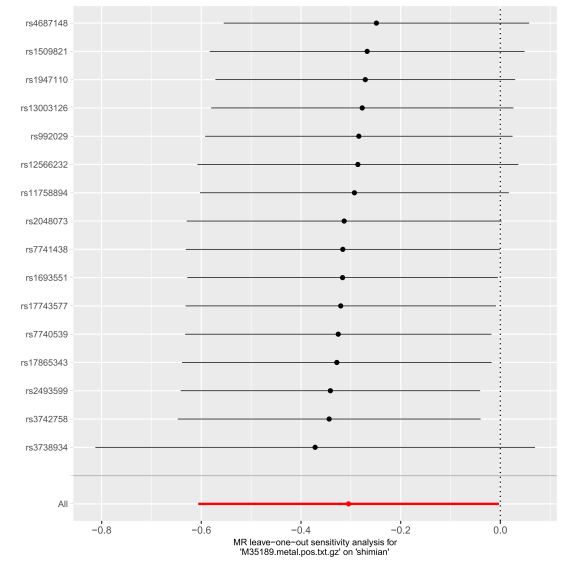

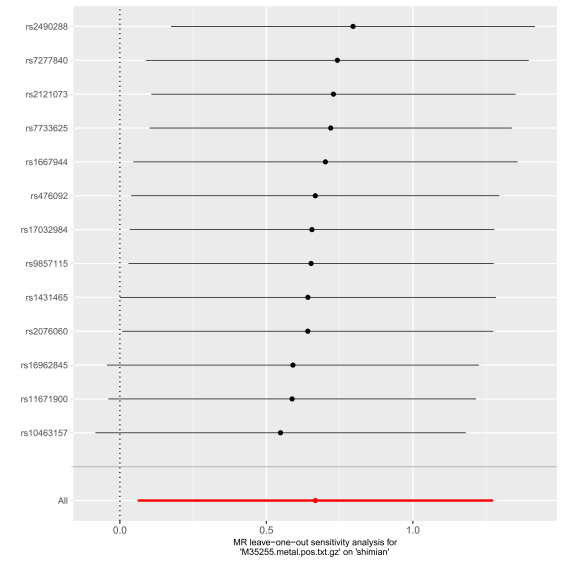

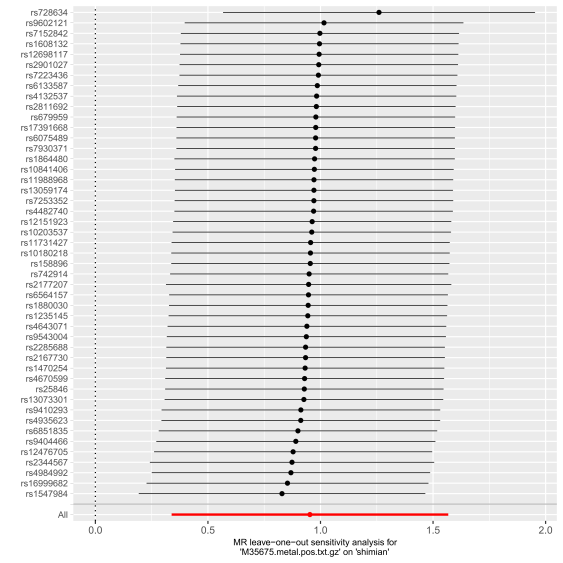

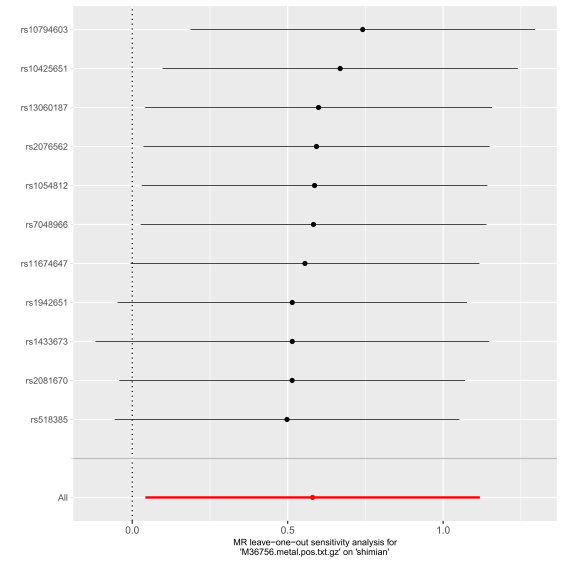


Supplementary Figure 1. The IS group used the leave-one method to analyze the results


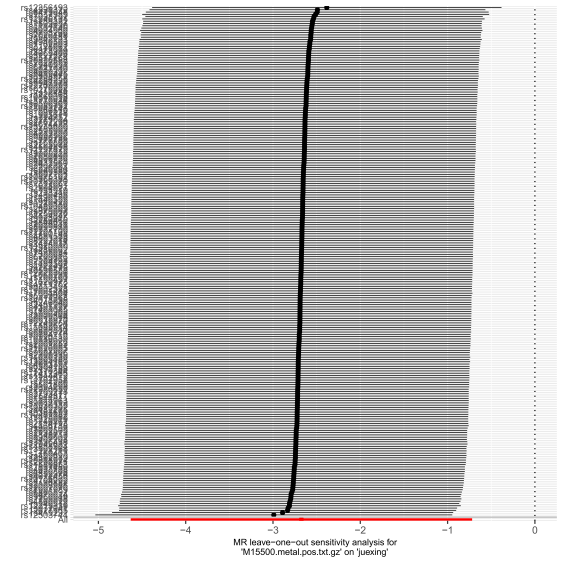

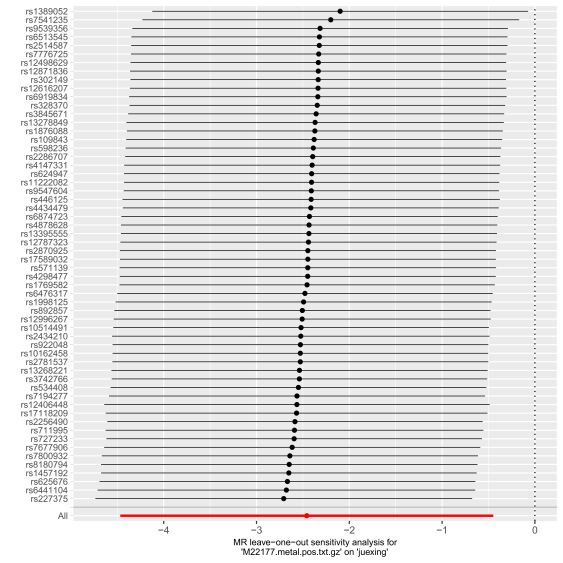

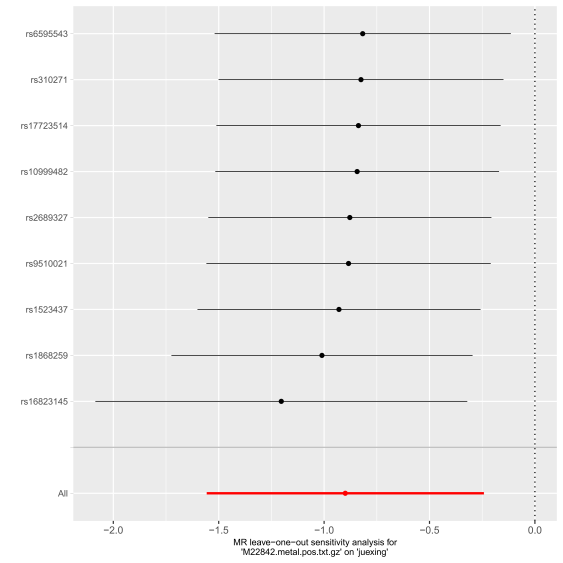

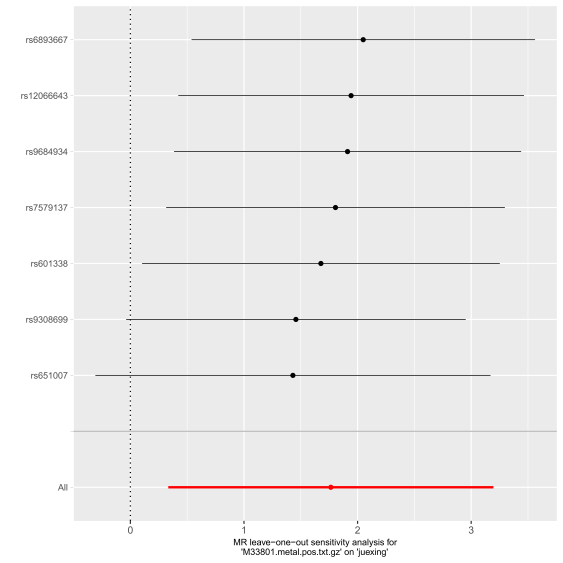

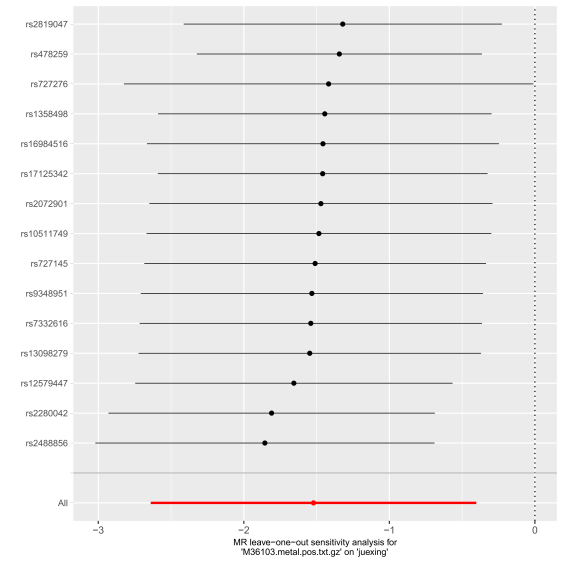

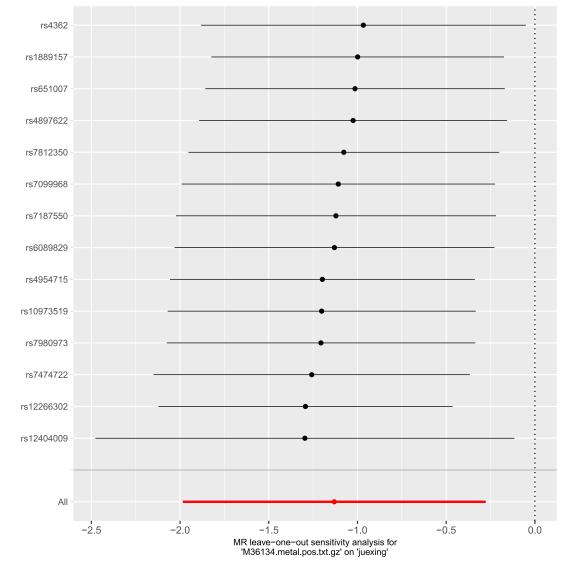

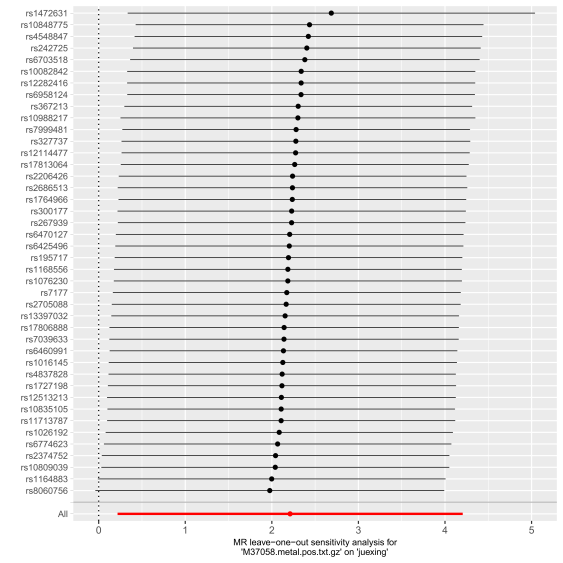


Supplementary Figure 2. The CRSWD group used the leave-one method to analyze the results
